# Editorial: Macrophages at the crossroads of fibrosis and immunosuppression

**DOI:** 10.3389/fimmu.2026.1968271

**Published:** 2026-09-22

**Authors:** Alessio Torcinaro, Alessio Reggio, Alessandro Palma

**Affiliations:** 1 Institute of Biochemistry and Cell Biology (IBBC), National Research Council of Italy (CNR), Rome, Italy; 2 Departmental Faculty of Medicine, Saint Camillus International University of Health Sciences, Rome, Italy; 3 Department of Biology, University of Rome “Tor Vergata”, Rome, Italy; 4 Division of Neuroscience, Department of Psychology, Sapienza University of Rome, Rome, Italy

**Keywords:** fibrosis, immune activation, immune system, immunosupperssion, inflammation, macrophages, myeloid cells

## 1 Introduction

Macrophages are increasingly recognized as central regulators of tissue remodeling and homeostasis rather than terminally polarized immune cells. Beyond their classical role in host defense and phagocytosis, macrophages dynamically integrate inflammatory, metabolic, stromal, and mechanical cues to orchestrate tissue remodeling and repair, immune responses, and intercellular communication. In pathological conditions, however, these adaptive programs become dysregulated, driving chronic inflammation, fibrosis, immunosuppression, and progressive tissue deterioration, ultimately leading to impaired tissue homeostasis.

Providing a comprehensive overview of the many functions of macrophages remains a challenging task. We therefore assembled a collection of complementary contributions from researchers actively advancing our understanding of macrophage biology. To this end, the Research Topic “Macrophages at the Crossroads of Fibrosis and Immunosuppression” brought together diverse perspectives that collectively reflect the current state and future directions of this rapidly evolving field.

At the time of its completion, the Research Topic had brought together 16 manuscripts involving 116 scientists from 52 institutions across 8 countries: Australia, China, Hungary, Italy, Japan, Poland, the United Kingdom, and the United States of America. This broad international participation underscores the widespread interest in macrophage biology and its relevance across diverse areas of biomedical research.

The relevance of this Research Topic is further highlighted by its broad scientific impact, with more than 41,000 topic views, 28,000 article views, and over 7,000 article downloads. These metrics reflect the growing interest of the scientific community in understanding macrophage-driven mechanisms linking fibrosis, immune regulation, and tissue remodeling.

## 2 Macrophage plasticity beyond the M1/M2 paradigm

A central theme emerging from this Research Topic is that the conventional M1/M2 paradigm provides an overly simplistic representation of macrophage biology. Several contributions highlight the inadequacy of this binary classification in capturing the remarkable heterogeneity and functional plasticity of macrophages. Instead, macrophage identity is increasingly understood as a dynamic and context-dependent continuum shaped by local microenvironmental signals, tissue-specific cues, and disease-associated stimuli. Macrophages therefore occupy a continuum of activation states characterized by overlapping inflammatory, reparative, metabolic, and immunosuppressive features (**Figure 1**).

**Figure 1:**
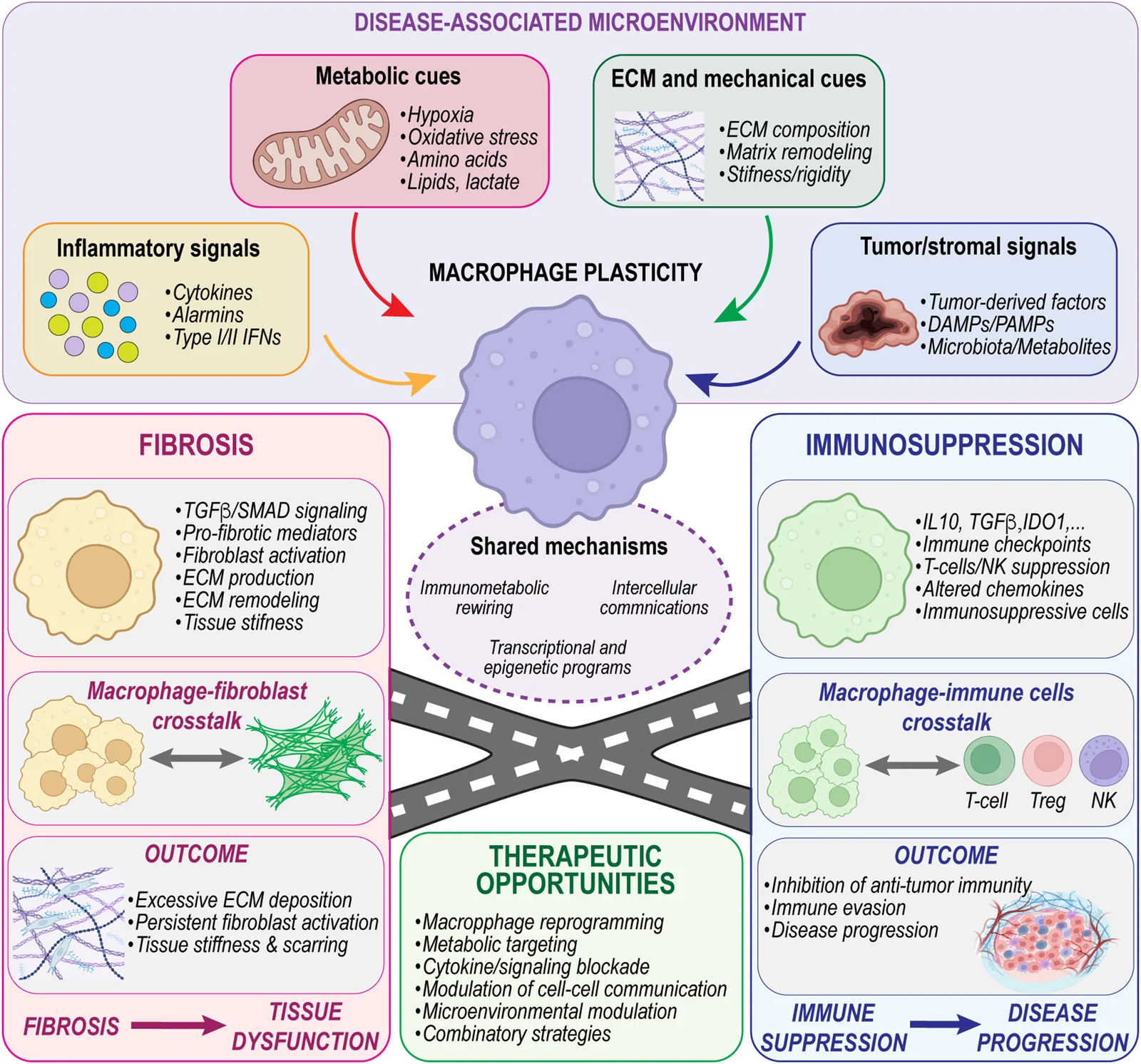
Conceptual framework of macrophage plasticity at the crossroads of fibrosis and immunosuppression. Disease-associated microenvironmental cues shape macrophage functional states through interconnected immunometabolic, transcriptional/epigenetic, and intercellular communication programs, ultimately contributing to fibrotic remodeling and immunosuppression and providing potential opportunities for therapeutic intervention.

In oncogenic settings, such as melanoma, stem-like tumor cells were shown to promote a non-canonical macrophage phenotype. This phenotype was characterized by the simultaneous activation of inflammatory, interferon-related, pro-angiogenic, and immune-regulatory programs, ultimately impairing NK-cell cytotoxicity and supporting tumor progression (1). Similarly, reviews focusing on fibrosis, skeletal muscle disease, and TREM2 biology emphasized that macrophage functional states are governed by highly interconnected transcriptional, metabolic, and epigenetic circuits that transcend simplistic polarization models (2–4). Within the central nervous system, the HMC3 *in vitro* microglial model further illustrated the remarkable adaptability of macrophage-like cells across neurodegenerative contexts, including oxidative stress, inflammasome activation, autophagy dysregulation, and phagocytic remodeling (5). Collectively, these studies support the concept that macrophage plasticity represents a multidimensional and environmentally encoded process. These observations suggest that macrophage plasticity is not merely a response to disease-associated signals, but an active process through which macrophages integrate multiple environmental cues to establish functional states that can either support tissue adaptation or contribute to pathology.

## 3 The microenvironment as a driver of macrophage states

Another major concept emerging from this Research Topic is the central role of tissue microenvironments in directing macrophage behavior. Fibrotic and tumor niches shape macrophage states through extracellular matrix remodeling, cytokine gradients, soluble mediators, metabolic signals, and intercellular communication networks. Using tunable three-dimensional collagen scaffolds, one of the studies included in this collection demonstrated that increased matrix stiffness influences macrophage phenotypes during co-culture with fibroblasts, promoting increased expression of CD54 and CD140a, both associated with inflammatory and fibrotic processes (6). These findings highlight the importance of mechanobiology in macrophage-fibroblast crosstalk and underscore the value of physiologically relevant three-dimensional systems. Similarly, tumor-derived miRNAs were shown to shape tumor-associated macrophage plasticity in breast cancer through extracellular vesicle-mediated communication, highlighting bidirectional signaling circuits that contribute to immune suppression, metastasis, and therapeutic resistance (7). Additional studies further underscored the role of macrophages as central hubs of intercellular communication by demonstrating a link between complement activation and M2-like macrophage accumulation during hepatic injury (8) and identifying TMEM106A-dependent chemokine signaling as a mechanism contributing to macrophage-mediated inflammation and foam cell formation in atherosclerosis (9). Importantly, these signals do not act independently but converge on intracellular transcriptional, metabolic, and signaling programs that determine macrophage functional states.

## 4 Immunometabolic and regulatory circuits of macrophage function

Several contributions also emphasized the increasingly recognized role of immunometabolism in shaping macrophage function. Rather than representing mere secondary consequences of activation, metabolic programs are now recognized as key determinants of macrophage identity and behavior. Reviews focusing on fibrosis, chronic obstructive pulmonary disease, and muscle regeneration described how glycolysis, oxidative phosphorylation, redox homeostasis, and lipid metabolism shape macrophage inflammatory and reparative responses through interconnected metabolic, epigenetic and transcriptional mechanisms (2–4, 10). Experimental work investigating NOX2 inhibition provided additional mechanistic insight into this concept by demonstrating that suppression of ROS generation reshapes inflammatory macrophage transcriptional programs, attenuating pro-inflammatory cytokine production while promoting pathways associated with anti-inflammatory and tissue-repair responses even under strong LPS stimulation (11). In neurodegenerative settings, carnosine-mediated protection of human microglia against Aβ oligomer-induced dysfunction further highlighted the close interplay between oxidative metabolism, energy homeostasis, and phagocytic competence (12). Together, these findings underscore metabolic rewiring as a key component of macrophage functional plasticity across diverse disease contexts.

## 5 Macrophages in fibrosis and immunosuppression: from disease-associated states to therapeutic opportunities

In fibrotic diseases, macrophages can acquire disease-associated states that actively contribute to persistent tissue remodeling rather than simply reflecting the inflammatory environment (13). Across different organs, fibrosis-associated macrophages display distinct transcriptional and functional programs shaped by tissue-specific cues, cellular origin, metabolic programs, and interactions with stromal cells. Rather than representing a uniform population, these macrophages may occupy distinct yet overlapping states associated with extracellular matrix remodeling, fibroblast activation, inflammatory persistence, and tissue repair. The apparently divergent effects of TREM2 across fibrotic organs further illustrate how the same macrophage-associated pathway can produce different outcomes depending on the cellular and tissue context (2, 4).

This context dependence has important implications for therapeutic intervention. Rather than broadly suppressing macrophage activity, an emerging therapeutic rationale is to selectively modulate disease-associated macrophage states or the signaling networks that sustain them, while preserving their physiological functions in host defense and tissue repair. The studies included in this Research Topic illustrate that such modulation can be pursued at different levels, from macrophage-intrinsic pathways to the extracellular signals that shape their functional states. Experimental modulation of inflammatory and metabolic pathways can alter macrophage functional states, as illustrated by the effects of DHJSD in intervertebral disc degeneration and NOX2 inhibition in inflammatory macrophages (11, 14) At the level of intercellular and upstream signaling, miRNA-mediated reprogramming of tumor-associated macrophages, complement pathway modulation, and IFN-γ blockade in macrophage activation syndrome further illustrate how macrophage-associated disease processes can be influenced by targeting the signaling networks that shape immune cell function (7, 8, 15). The experience of idiopathic pulmonary fibrosis further illustrates this shift in therapeutic thinking: the abandonment of broad immunosuppressive regimens in favor of approaches directed toward the cellular and molecular drivers of fibrosis underscores the importance of targeting disease mechanisms rather than suppressing immune activity indiscriminately (16).

Altogether, these contributions support a therapeutic framework in which macrophage function is modulated according to disease, tissue, and microenvironmental context, rather than through indiscriminate alteration of macrophage activation.

## 6 Translational challenges and future directions

Translating this context-dependent therapeutic framework into clinical practice, however, remains challenging. Rather than broadly depleting or suppressing macrophages, effective therapeutic strategies will need to selectively modulate disease-associated states while preserving their essential functions in host defense, tissue repair, and homeostasis. This is particularly challenging because macrophage states are highly plastic, tissue-specific, and dynamically influenced by local microenvironmental signals, making it difficult to define universally applicable therapeutic targets. Moreover, macrophage populations identified in experimental models may not fully recapitulate the cellular states present in human disease.

This complexity is further increased by biological variables that influence macrophage identity and function beyond disease-associated stimuli. Age, sex, developmental origin, and tissue identity can shape macrophage transcriptional, metabolic, and functional programs, contributing to differences in macrophage responses across tissues and individuals (17–19). These variables should therefore be considered when interpreting macrophage phenotypes and evaluating the translational relevance of candidate therapeutic targets.

Addressing these challenges will require approaches capable of resolving macrophage states within their native tissue context and across disease progression. Recent advances in single-cell RNA sequencing, spatial transcriptomics, integrated multi-omics, and high-dimensional imaging are providing increasingly detailed maps of macrophage heterogeneity and tissue organization. When combined with functional and longitudinal analyses, these approaches may help distinguish disease-associated macrophage programs from transient activation states and identify the molecular circuits that can be selectively targeted. Such strategies may ultimately enable a transition from descriptive macrophage classification toward context-aware therapeutic intervention.

The ultimate challenge will be to translate this increasing resolution of macrophage biology into interventions that are sufficiently selective to modify pathogenic states without disrupting the physiological functions required for tissue homeostasis.

## 7 Conclusions

Taken together, the contributions assembled in this Research Topic reinforce a view of macrophages as dynamic regulators of tissue ecosystems rather than fixed inflammatory or immunosuppressive populations. Their functional states emerge from the integration of microenvironmental, metabolic, mechanical, and stromal cues and can contribute to either tissue adaptation and repair or persistent inflammation, fibrosis, and immunosuppression. This context dependence places macrophages at a critical interface between tissue remodeling and immune regulation, while also creating opportunities for therapeutic intervention. The challenge ahead is therefore not simply to classify macrophage states, but to define the molecular and environmental circuits that establish them and to translate this knowledge into selective, context-aware interventions that restore tissue homeostasis without compromising essential macrophage functions.
